# Genomic Identification, Evolution and Sequence Analysis of the Heat-Shock Protein Gene Family in Buffalo

**DOI:** 10.3390/genes11111388

**Published:** 2020-11-23

**Authors:** Saif ur Rehman, Asif Nadeem, Maryam Javed, Faiz-ul Hassan, Xier Luo, Ruqayya Bint Khalid, Qingyou Liu

**Affiliations:** 1 State Key Laboratory for Conservation and Utilization of Subtropical Agro-Bioresources, Guangxi University, Nanning 530005, China; saif_ali28@yahoo.com (S.u.R.); luoxier92@163.com (X.L.); 2 Department of Biotechnology, Virtual University of Pakistan, Lahore-54000, Pakistan; asif.nadeem@vu.edu.pk; 3 Institute of Biochemistry and Biotechnology, University of Veterinary and Animal Sciences, Lahore-54000, Pakistan; Maryam.javed@uvas.edu.pk (M.J.); ruqiakhalid0@gmail.com (R.B.K.); 4 Institute of Animal and Dairy Sciences, Faculty of Animal Husbandry, University of Agriculture, Faisalabad-38040, Pakistan; f.hassan@uaf.edu.pk

**Keywords:** HSP, physiochemical properties, multiple sequence alignment, phylogenetic analysis, *Bubalus bubalis*

## Abstract

Heat-shock proteins (HSP) are conserved chaperones crucial for protein degradation, maturation, and refolding. These adenosine triphosphate dependent chaperones were classified based on their molecular mass that ranges between 10–100 kDA, including; *HSP10*, *HSP40*, *HSP70*, *HSP90*, *HSPB1*, *HSPD*, and *HSPH1* family. HSPs are essential for cellular responses and imperative for protein homeostasis and survival under stress conditions. This study performed a computational analysis of the HSP protein family to better understand these proteins at the molecular level. Physiochemical properties, multiple sequence alignment, and phylogenetic analysis were performed for 64 HSP genes in the *Bubalus bubalis* genome. Four genes were identified as belonging to the *HSP90* family, 10 to *HSP70*, 39 to *HSP40*, 8 to *HSPB*, one for each *HSPD*, *HSPH1*, and *HSP10*, respectively. The aliphatic index was higher for *HSP90* and *HSP70* as compared to the *HSP40* family, indicating their greater thermostability. Grand Average of hydropathicity Index values indicated the hydrophilic nature of *HSP90*, *HSP70*, and *HSP40*. Multiple sequence alignment indicated the presence of highly conserved consensus sequences that are plausibly significant for the preservation of structural integrity of proteins. In addition, this study has expanded our current knowledge concerning the genetic diversity and phylogenetic relatedness of HSPs of buffalo with other mammalian species. The phylogenetic tree revealed that buffalo is more closely related to *Capra hircus* and distantly associated with *Danio rerio*. Our findings provide an understanding of HSPs in buffalo at the molecular level for the first time. This study highlights functionally important HSPs and indicates the need for further investigations to better understand the role and mechanism of HSPs.

## 1. Introduction

Heat-shock proteins (HSP) are well-known and highly conserved protein families responsible for cellular responses to various stresses. These evolutionary conserved molecular chaperones play a vital role in the survival of organisms in response to elevated ambient temperatures. Protection from cellular damage, alleviation of stresses such as energy depletion, cytokines, ischemia, and development of thermotolerance are major physiological processes controlled by HSP [1]. The pivotal role of HSP in cellular responses under stress conditions and normal physiological activities is well established [2]. HSP were first reported by the Ferruccio Ritossa in 1962 during his experiment on the *Drosophila* at the Genetics Institute in Pavia [3].

The HSP are highly conserved, ubiquitous, and functional proteins found in all organisms, starting from bacteria to humans. It has been suggested that HSP play an essential physiological role in normal situation as well as in cellular and systematic stresses, especially in farm animals, including cattle and buffalo [1]. Nonetheless, the most crucial function of HSP is the protection of cells from heat shock through resisting against denaturation of cellular proteins [4]. The cellular response is one of the primary pathways which help the animal to endure thermal stress. This pathway assists animals in surviving several anomalies in the functioning of cells. HSP is an important end product of cellular and tissue defense mechanism and expression of HSP gets markedly elevated during heat shock [5]. Various physiological and physical stressors are responsible for the activation of HSP proteins [6]. Elevation of HSP response can help to improve the thermotolerance of bovines [7]. Heat stress is one of the major factors affecting livestock production across the world. HSPs not only develop thermotolerance in livestock species including dairy cattle but also considered to be potential biological marker to quantify the magnitude of heat stress across different livestock species [8]. Several studies have proved the importance of HSPs in regulating various types of immunity [9]. It has been reported that the addition of HSP-derived peptides in lymphocytes of buffalo helped in increasing adaptive and innate immune effectors [9]. HSPs have also been associated with reproductive efficiency. For instance, in Holstein cows, differences in HSP40 genes have been correlated with early embryonic development in vitro. Similarly, polymorphism in HSP70 has been reported to be associated with difference in reproductive performances of *Bos indicus* breeds [10].

These proteins also act as molecular chaperones for various other cellular proteins, actively involved in multiple regulatory pathways, and have strong cytoprotective properties [2]. As a molecular chaperon, HSP prevent the inappropriate aggregation of proteins and regulates the final packaging, degradation, and/or repair of nascent polypeptides. HSP plays an essential role in maintaining homeostasis at cellular levels due to their specialized functions, particularly under heat stress conditions. HSP contribute significantly to the protection of cellular damage and other adverse effects of heat stress in animals. Failure of HSP to sustain their normal physiology can lead to poor or no thermotolerance rendering animals a victim of heat stress under higher ambient temperatures [11].

HSP have been classified into various families depending upon their molecular weights. HSP have been classified as *HSP40*, *HSP60*, *HSP70*, *HSP90*, *HSP100* and small *HSPs* [12]. These HSP families are known for their distinct roles in different biological processes. For instance, various pathological conditions such as viral infections, cancer, muscular dystrophy, and neurodegeneration have been associated with *HSP40* [13]. Similarly, *HSP70* after attaching with protein substrate, assist in transportation as well as unfolding. Moreover, *HSP70* has been involved in different pathological disorders such as infectious diseases, cancer, and neurodegenerative problems. *HSP90* is known to activate certain oncogenic proteins through regulation of their conformation and stability. The inhibition of HSP90 could suppress such oncogenic signaling pathways. Due to the association of HSPs with certain diseases, these are potential targets for designing and developing therapies for these ailments [14,15].

Water buffalo is an important livestock species and second-best milk producer mammal after cattle (by contributing 5% of the total global milk production) distributed in different areas of tropics and subtropics [16,17]. Owing to diverse crucial role of HSP in cellular physiology, genomic characterization, and sequence analysis is imperative to better understand the evolutionary relationship, physiochemical properties, and chromosomal distribution in the buffalo. Our present study aimed to perform the HSP gene family analysis at the genomic level. Our findings provide insights into the understanding of the genomic architecture of the HSP family in buffalo and provide key evidence for further functional studies.

## 2. Materials and Methods

### 2.1. Identification of the HSP Genes in Buffalo

Published sequences from *Bos taurus* were used as queries for the genome-wide identification of HSP genes in the buffalo, so we used representative sequences of *HSP10* (NP_776771.1), *HSP40* (XP_005224490.1), *HSP70* (NP_976067.3), *HSP90* (NP_001012688.1), *HSPB* (NP_001020740.1), *HSPD* (NP_001160081.1), and *HSPH1* (NP_001068770.1) from *Bos taurus*. We analyzed HSP protein sequences using Protein-protein basic local alignment (BLAST) search with an E value equal or less than 1.0 × e^−5^ of the non-redundant protein sequences in buffalo using all the parameters by default. After retrieving the required sequences, redundancy of the sequences was checked to avoid ambiguity. Chromosome locations of each HSP gene were obtained from buffalo genome resources. The corresponding gene positions files were obtained from the buffalo genome annotation file with the general feature format (GFF), which can be set as the input files of the MCScanX program, as reported previously [18]. Moreover, the exon-intron structure was analyzed by the buffalo genome annotation file using the in-house scripts. The conserved protein domains in buffalo HSP families were blasted against the conserved domain database (CDD) from NCBI.

### 2.2. Characterization of Physiochemical Properties of HSP Genes

To characterize the physical and chemical parameters of HSP proteins, ProtParam tool (https://web.expasy.org/protparam/) was used [19]. Protein sequences were submitted to identify the molecular weight (MW), number of amino acids, isoelectric point (pI), aliphatic index (AI), instability index (II) and grand average of hydropathicity (GRAVY). Moreover, conserved protein motifs of HSP were analyzed by using the Multiple Expectation Maximization for Motif Elicitation (MEME) programs with a maximum of 10 motifs [20]. Three-dimension protein structure for various HSP protein (*HSP10*, *HSP40*, *HSP70*, *HSP90*, *HSPB1* and *HSPH1*) were constructed by using Phyre2 software (http://www.sbg.bio.ic.ac.uk/phyre2) with fold recognition end homology modeling for the identification of secondary structure attributes.

### 2.3. Phylogenetic Analysis

Results of multiple sequence alignment were further used for phylogenetic analysis using MEGA7 [21]. The evolutionary history was inferred by using the Maximum Likelihood method based on the Jones-Taylor Thornton (JTT) matrix-based model [22]. The tree with the highest log likelihood (−581.71) was selected. The percentage of trees in which the associated taxa clustered together is shown next to the branches. Initial tree(s) for the heuristic search were obtained automatically by applying Neighbor-Join and BioNJ algorithms to a matrix of pairwise distances estimated using a JTT model with 1000 bootstrap resampling, and then selecting the topology with superior log likelihood value. The tree is drawn to scale, with branch lengths measured in the number of substitutions per site. The analysis involved 77 amino acid sequences. All positions containing gaps and missing data were eliminated. There was a total of 16 positions in the final dataset. Evolutionary analyses were conducted in MEGA7 [21].

### 2.4. Sequence Analysis

Accession no of all sequences which were used for multiple sequence alignment and subsequent analyses are presented in Tables S1–S5. Comparison of buffalo HSP was carried out with other mammalian species including cattle (*Bos taurus*), goat (*Capra hircus*), sheep (*Ovis aries*), horse (*Equus caballus*), camel (*Camelus ferus*), mouse (*Rattus norvegicus*), pig (*Sus scrofa*), zebra fish (*Danio rerio*), mouse (*Mus musculus*), and dog (*Canis lupus familiaris*). Sequence homology of buffalo HSP family was carried out along with elucidation of phylogenetic relationships using the Geneious version 2020.1 created by Biomatters (https://www.geneious.com).

### 2.5. Identification of Single Nucleotide Polymorphism (SNP)

Multiple sequence alignment of coding regions of all the members of two major HSP gene families (*HSP90* and *HSP70*) in buffalo and *Bos taurus* were perused in DNASTAR Lasergene software to identify the number of synonymous and non-synonymous SNPs in buffalo [23]. Moreover, we also calculated the number of non-synonymous substitutions per non-synonymous site (Ka)/ the number of synonymous substitutions per synonymous site (Ks) ratio as described previously [24].

## 3. Results

### 3.1. Identification of HSP Gene Families and Their Physiochemical Properties

In the present study, we applied a comprehensive strategy to characterize the HSP in the buffalo genome. By using human and mouse sequences as queries, we detected four genes belonging to *HSP90*, 10 genes to *HSP70*, 39 genes to *HSP40*, 8 genes to *HSPB*, and one gene to each *HSPD*, *HSPH1*, and *HSP10* family. Buffalo each HSP gene family tree and percentage of identity with other species is represented in Figures S1–S7. The physical and chemical properties of these HSP genes in buffalo are presented in Table 1, Table 2, Table 3 and Table 4. The functional diversity of HSP isoforms could be realized from a wide range of their MW (10 kDa to 254 kDa) and the total number of amino acids in different HSP genes that ranged from 102 (*HSP10*) to 2223 amino acids in DnaJ homolog subfamily C member 13 (*DNAJC13*).

The results of the present study revealed that all the members of the *HSP90* family are unstable according to instability index. The isoelectric point indicates the acidic nature of protein members except for *TRAP1*, which is basic in nature. An AI greater than 65 indicates the thermostability of *HSP90* protein members whereas, lower GRAVY values of *HSP90* indicates its hydrophilic nature (Table 4). *HSP70* members are thermostable, acidic, and hydrophilic in nature. The instability index indicates the unstable nature of *HSPA14*, *HSPA4*, and *HSPA4L* protein members (Table 1). Various physical and chemical parameters of *HSP40* protein members are shown in Table 2. *HSP40* is the largest HSP gene family, with 39 genes widely distributed over different buffalo chromosomes with MW ranging from 15 kDa to 254 kDa. The largest gene of this family is *DNAJC13*, which harbors 59 exons, 2243 amino acids, and MW of 254 kDa. The details about the physiochemical nature of small HSPs, including *HSP10*, *HSPB*, *HSPD*, and *HSPH1*, are given in Table 5. All members of *HSPB* are thermostable except *HSPB7* and 8.

### 3.2. Phylogenetic Analysis

**Figure 1:**
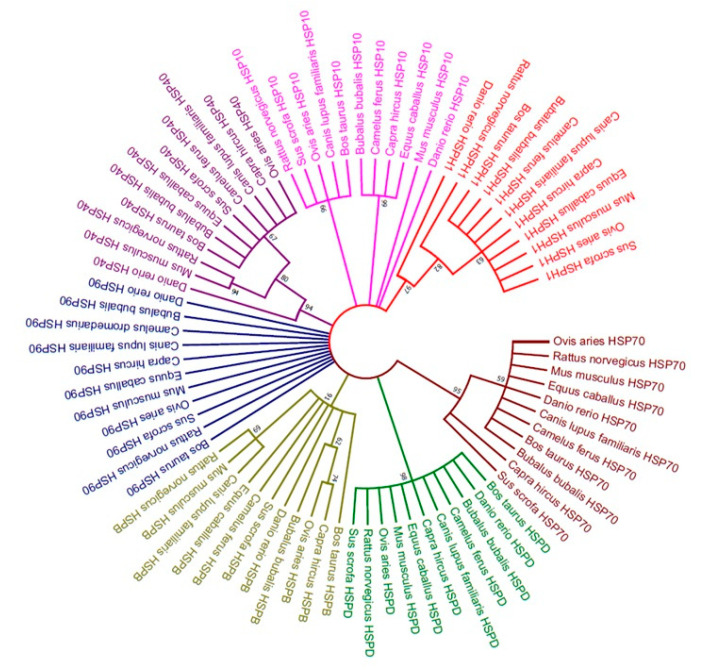
Molecular phylogenetic tree of heat-shock proteins (*HSP40*, *HSP70*, *HSP90*, *HSPB*, *HSPD*, *HSP10*, and *HSPH1*).

**Figure 2:**
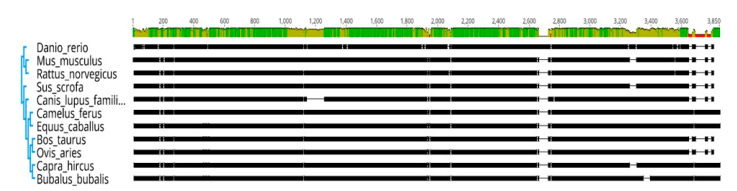
Phylogenetic tree with alignment view of *HSP40* (sequence 1–493), *HSP70* (494–1137) and *HSP90* (1138–1870), *HSPB* (1871–2087), *HSPD* (2088–2662), *HSPH1* (2663–3588) and *HSP10*KD (3589–3850). Percentage of sequence identity is represented with different colors in identity bar at top of the sequences green color indicates 100% identity, green-brown shows at least 30 and under 100% while red representing below 30 identity.

The evolutionary history was inferred by using the Maximum Likelihood method based on JTT matrix-based model. The bootstrap consensus tree inferred from 1000 replicates was taken to represent the evolutionary history of the taxa analyzed. Each HSP family genes clustered together in one group, revealing seven groups (Figure 1). Overall phylogenetic relationships revealed that buffalo HSP gene family is more closely related to *Bos taurus*, goat and sheep along with the common clade of horse and camel (Figure 2). Moreover, buffalo is distantly related to other mammals, including pig, dog, rat, mouse, and zebra fish. 

### 3.3. Gene Structure and Motif Analysis

**Figure 3:**
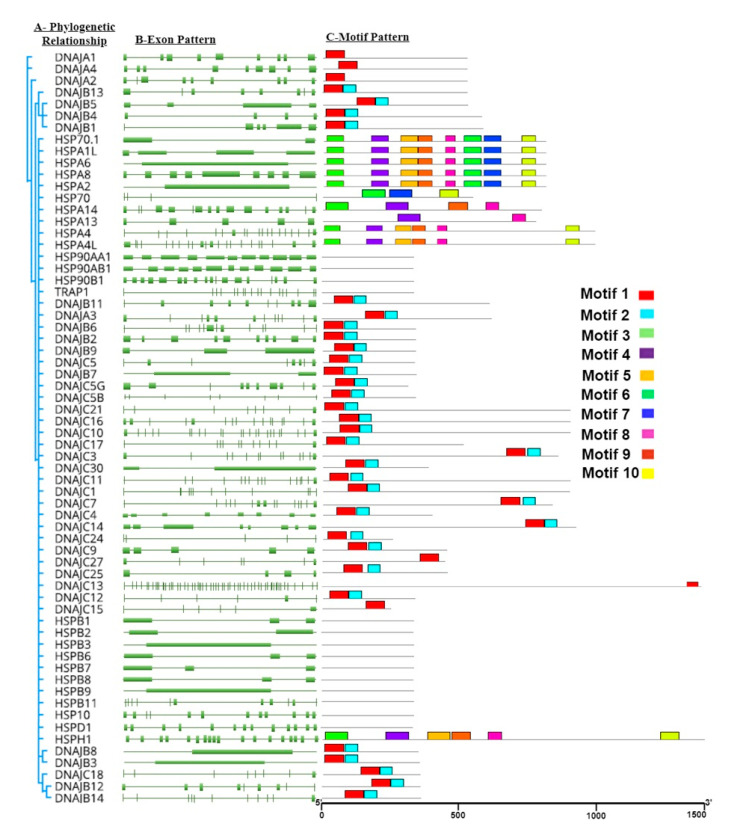

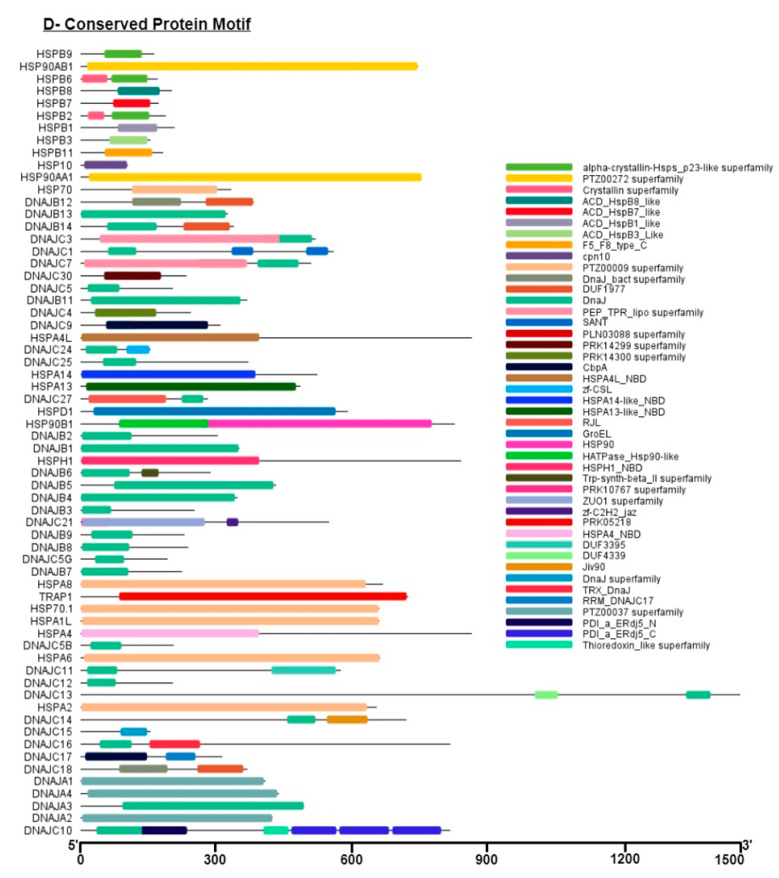
Phylogenetic relationships, exon motif patterns, and conserved protein motif of buffalo HSP gene family. (**A**) Phylogenetic tree of 64 buffalo HSP proteins. (**B**) Exon pattern of buffalo HSP. (**C**) Motif pattern of buffalo HSP proteins gene family. Ten putative motifs are indicated in different colored boxes. For details of motifs, refer to Table 5. (**D**) Conserved protein motif.

To gain further insight of HSP genes in buffalo, gene structures and conserved motifs were predicted. The number of exons varies in all genes; for instance, *DNAJC13* has a higher number of exons i.e., 59. The exonic pattern of buffalo HSP genes are shown in Figure 3B. Ten putative motifs of heat-shock protein gene family were observed in buffalo (Table 5). MEME motif 1 and 2 (consisting of 41 and 28 amino acid) were annotated as the *DnaJ* domain in Pfam search, while 3 to 9 motifs were annotated as *HSP70*. These motifs were also confirmed from NCBI CDD. Moreover, MEME was used to predict conserved motifs present within HSP families in the buffalo (Figure 3D).

**Figure 4:**
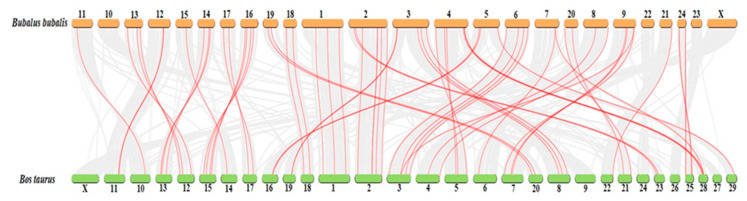
Collinearity analysis showing distribution of HSP genes in buffalo and cattle.

Collinearity analysis showed that HSP genes were distributed over 20 chromosomes in buffalo, while in cattle, these genes were randomly distributed over 25 chromosomes (Figure 4). Most of the genes were present on proximal or distal ends of chromosomes in buffalo.

### 3.4. HSP Protein Structural Configuration

**Figure 5:**
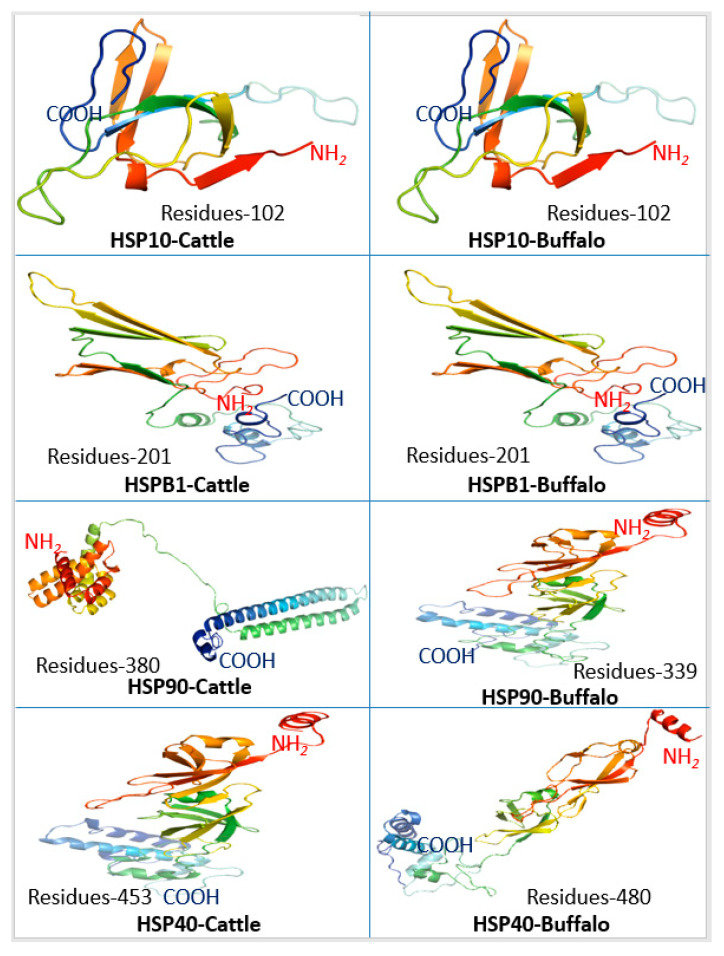
Three-Dimensional protein configuration of Heat-Shock Proteins in *Bos taurus* and buffalo.

Three-dimensional protein models were also predicted for various heat-shock proteins (*HSP10*, *HSPB1*, *HSP90*, *HSP40* and *HSP70*) in *Bos taurus* to elucidate comparative structural configuration (Figure 4). It was observed that the overall structure of *HSP10* in both species was unaltered with the same number (102) of amino acid residues. Similarly, in the case of *HSPB1*, no significant structural variation was observed among both species in 201 amino acid residues. For *HSP90*, considerable variation in the structure of the protein was observed in both species (Figure 5). In cattle, 380 residues were present, but in buffalo, only 339 amino acids were observed in final protein structure (Figure 5). Moreover, secondary structural elements also varied in both species as β-sheets observed in buffalo were absent in the case of *Bos taurus*. In cattle, protein was majorly consisting of α-helix. Similarly, the *HSP40* gene also showed structural variation in buffalo as the final structure comprised of 480 amino acid residues as compared to 453 in cattle (Figure 5). However, the structure of *HSP70* protein was similar in both species having amino acids 641.

### 3.5. SNP Analysis

Mutation analysis of 14 genes belonging to *HSP70* and *HSP90* families from buffalo and *Bos taurus* were compared to detect synonymous and non-synonymous SNPs. We identified a total of 194 SNPs in the *HSP90* family genes among which only 19 were non-synonymous (Table 6). The highest number of SNPs (128) was observed in the *TRP1* gene, among which 17 were non-synonymous. Moreover, the *TRP1* gene also exhibited a single indel of 3 nucleotides at position 1703–1705. *HSP90AA1* showed a total of 23 synonymous SNPs and only one non-synonymous SNP, while *HSP90AB1* and *HSP90B1* exhibited only 22 and 19 synonymous SNPs, respectively (Table S6).

The present study identified 291 SNPs in the *HSP70* family genes, among which 59 were non-synonymous. The highest number of SNPs (80) was observed in the *HSPA8* gene exhibiting 8 non-synonymous substitutions. The lowest number of SNPs (10) was detected in the *HSPA14* gene with two non-synonymous SNPs. *HSP70* gene showed highest non-synonymous to synonymous ratio by exhibiting 23 non-synonymous SNPs in a total of 62. Interestingly, the *HSPA2* gene of the *HSP70* family showed an indel of 15 nucleotides at position 777–791 (Table S6).

## 4. Discussion

HSP play crucial functions, including stress tolerance, cell survival, protein refolding, and prevention of protein denaturation. Moreover, HSP also facilitate the regulation of specific cellular processes such as signal transduction, protein synthesis, protein trafficking, and DNA replication [4,25,26]. It is well established that *HSP90* plays pivotal role in controlling cell cycle, transport, folding, and degradation of proteins as well as thermotolerance and induction of signal transduction. Studies have revealed that *HSP90* is present in various organisms and this HSP family is highly conserved [27]. HSPs are the molecular chaperones that help to maintain activity of cells in different stressful states. Some of these proteins are produced due to different stressful stimuli (inducible), while others are expressed constitutively. Cellular metabolism, ultraviolet radiations, alteration in protein structures, cytotoxic drugs, and heat are some of the stress stimuli that usually induce HSP expression. These proteins are highly involved in facilitating the degradation of misfolded proteins and regulating proper protein folding [28]. In this study, we analyzed a total of 64 HSP genes in buffalo. Members of *HSP90* are found to be present on four chromosomes in buffalo similar to *Bos taurus* [29], suggesting that these protein molecules are highly conserved and share a common ancestor (Figure S3).

### 4.1. Physiochemical Properties of HSP Gene Families

The characterization of the physiochemical properties of proteins of various gene families is essential to identify the functions and properties of proteins encoded by these genes. Isoelectric point indicates that members of *HSP90* in buffalo are acidic in nature (pI < 7.0), except for the *TRAP1* gene, which was basic in nature similar to human *HSP90* [30].

AI of the globular proteins is associated with their thermostability [31]. The AI, the respective volume of a protein occupied by aliphatic side chains (alanine, valine, isoleucine, and leucine), may be interpreted as a positive factor for increasing thermostability of globular proteins [31]. As the AI is associated with the thermostability of globular proteins, so a higher value of the AI reveals the high thermostability of protein. Hence, a higher AI of HSP90 and HSP70 showed that members of these proteins are more thermostable compared to HSP40 members. Similar results have also been observed in cattle genome [29]. In the present study, the *HSP90* gene family of buffalo had AI greater than 65, indicating the stability of proteins at high temperatures. Similarly, the higher expression of *HSP90* has been found in goat during the peak summer season, revealing the extent of thermostability of HSPs [32].

The GRAVY value is used to predict water and protein interaction. The GRAVY value for a protein or a peptide is defined as the sum of hydropathy values of all amino acids divided by the protein length. GRAVY qualifies and quantifies the hydrophobic or hydrophilic property of a protein. A positive GRAVY score indicates a globally hydrophobic protein, whereas a negative GRAVY score is related to hydrophilic protein [33]. Hence, more the negative GRAVY score, more soluble the protein is. All members of HSP40, HSP70, and HSP90 had a negative GRAVY score in the present study, suggesting these all members are soluble proteins. However, highly negative GRAVY values of *HSP90* observed in the present study indicate the greater hydrophilic nature of its members. It can help to enhance the functional properties of protein binding and oligomerization [34,35]. Among heat-shock proteins, *HSP90* is considered an essential and highly conserved molecular chaperone widely present in eukaryotic cells [36]. *HSP90* in buffalo share high sequence similarities with other species (Figure S3). The presence of conserved motifs helps interact with other proteins and increases their chaperone function [37,38].

A total of 10 *HSP70* members were observed in buffalo. Interestingly, all members of *HSP70* showed acidic nature and almost similar isoelectric points indicating the possibility of functional similarity among buffalo *HSP70* members. It has been suggested earlier that *HSP70* has conserved biological functions [39]. GRAVY values of *HSP70* protein of buffalo indicates their hydrophilic nature and high values of AI revealed their thermal stability in a wide range of temperatures. These findings confirm the consistency of its chaperoning role in protection against specific cellular and systemic stresses that can lead to protein denaturation [40]. In evolution, *HSP70* is the most conserved protein, and its conserved domains and functions are considered responsible for across species conservation [11,41]. In the present study, a tree with an alignment view of *HSP70* showed the presence of conserved domains. Buffalo is closely related to *Bos taurus*, followed by goat and sheep (Figure S2). The comparative analysis of the *HSP70* protein in water buffalo revealed that most of the sequences were conserved across species. Comparative sequence analysis for bubaline *HSP70.1* indicated the high similarity of genes among mammalian species, such as cattle, goat, human, mouse, and pig, respectively. Furthermore, phylogenetic analysis of bovine and bubaline *HSP70.1* gene with other species revealed Indian zebu cattle grouped close to *Bos taurus* followed by buffalo and goat, while porcine, ruminants, and murine formed distinct cluster [42,43].

Heat-shock protein 40 (*HSP40/DnaJ*) is evolutionary conserved and thought to play an essential role in translocation, degradation, translation, folding, and unfolding of proteins by stimulating the ATPase activity of *HSP70* protein chaperones [44,45]. All members of *HSP40* have domain J, which helps in binding with *HSP70*. Also, 41 *DnaJ* domains have been reported in humans [46]. In the present study, 39 putative members of *HSP40* were found. GRAVY value of *HSP40* members represents the hydrophilic nature of these proteins. Furthermore, pI values of *HSP40* protein members suggest that some of these proteins possess acidic nature while other proteins appear to be basic. It may explain the different functional properties of these proteins and provides insights for designing further experiments to elucidate their functional diversity. *HSP40* represents a large and diverse protein family and is usually divided into three classes [47]. Type I *HSP40s* is known to have a glycine and phenylalanine (G/F)-rich region and a cysteine-rich, zinc finger-like region (ZFLR). Type II *HSP40s* only contain a G/F-rich region, while type III *HSP40s* have neither a G/F-rich region nor a ZFLR [48]. Comparative sequence and phylogenetic analyses revealed that the *HSP40* family of buffalo is closely related to *Bos taurus* (Figure S1). Another study conducted on the bovine genome also indicated the presence of evolutionary conserved residues in *HSP40* [29].

### 4.2. Sequence Analyses

Data of protein sequences can provide useful information regarding evolutionary conserved regions important for various biological functions. Multiple sequence alignment is one of the ways to identify these conserved constraints and to compile data for structural and functional analysis of proteins [49]. To investigate the protein sequence features of the heat-shock proteins, 10 motifs of HSP gene families in buffalo were predicted by the MEME tool, and the regular expression levels of the conserved motifs are listed in Table 5. These conserved motifs can play a crucial role in the structural and functional organization of proteins. Similarly, another study on *Ciona savignyi*, identified conserved motifs in *HSP60*, *HSP70*, and *HSP90* by using MEME [50].

### 4.3. Phylogenetic Analysis

Molecular phylogenetic tree of heat-shock proteins (*HSP40*, *HSP70*, *HSP90*, *HSPB*, *HSPD*, *HSP10*, and *HSPH1*) was constructed by using the Maximum Likelihood method based on the JTT matrix-based model [22]. The bootstrap consensus tree inferred from 1000 replicates was taken to represent the evolutionary history of the taxa analyzed (Figure 1). Overall phylogenetic relationships revealed that buffalo is more closely related to goat than *Bos taurus*, and sheep along with horse and camel (Figure 2). Moreover, buffalo is distantly related to other mammals, including pig, dog, rat, mouse and zebra fish. A study conducted on cattle also revealed similar results as phylogenetic analysis of *HSPA8* and *HSPA1A* indicted the high similarity between buffalo and sheep [51]. Previously, molecular characterization of *HSP70.1* of goat have shown that buffalo, cattle, sheep, and goat share a common ancestor whereas human, horse and pig showed dissimilarities thus having different ancestors [52]. Moreover, Karan Fries cattle was evolutionary related to *Bos taurus*, *Bos indicus*, and buffalo, whereas distinctly related to rat and mouse [53].

### 4.4. Protein Structural Configuration

Three-dimensional (3-D) protein configurations of HSP in *Bos taurus* and buffalo was predicted. The analysis of 3-D secondary protein structures revealed the structural variations only in *HSP90* and *HSP40* of *Bos taurus* and buffalo, while other HSPs were similar or structurally related in both species. These findings confirm the results obtained in gene structure and phylogenetic analysis that HSPs are conserved proteins. Eukaryotic *HSP90* proteins had a modular structure with an N-terminal ATP binding domain connected to a middle domain by a ‘linker’ of variable length. The N-terminal and middle domains together form a “split” ATPase site that is also the binding pocket for GA [54]. *HSP40* can function as a molecular chaperone to bind non-native polypeptides. *HSP40* pairs with *HSP70* to achieve its function of protein folding, transport, and degradation functions. The J-domain of *HSP40* may stimulate the ATPase activity of *HSP70* and the C-terminal peptide-binding fragment of *HSP40* can load the bound non-native polypeptide to *HSP70* [55]. *HSP70* and *HSP40* are essential chaperons affecting the protein folding in the cell. Three-dimensional structural configurations of *HSP40* in cattle and buffalo varied in number of amino acid residues and overall secondary structural attributes that may be associated with differential chaperon functions and stress responses in both species. Further investigations at the transcriptome level are required to corroborate these findings.

### 4.5. SNP Analysis

The present study revealed that *HSP90AA1*, *HSP90AB1* and *HSP90B1* are more conserved genes than TRP1 due to a higher number of non-synonymous SNPs in *TRP1*. Similarly, our findings indicate that *HSP70.1, HSPA14*, and *HSPA1L* are more conserved than other genes of *HSP70* family. We observed a relatively higher ratio of non-synonymous to synonymous SNPs in the *HSP70* gene (0.59) as compared to other genes of the *HSP70* family. It indicates that *HSP70* gene is under selection owing to its strong association with cellular thermotolerance [1]. Moreover, the expression dynamics of the *HSP70* gene are used as a potential biomarker to reveal the level of heat stress and the ability of animals to withstand climate stress [56]. It is mainly attributed to the fact that HSP expression indicates cellular response and intensity of stress in animals [57]. At the transcription level, persuaded expression of *HSP70* genes is coordinated as upstream elements at promoter region directly control the expression profiles of *HSP70*. Studies have shown a higher expression of HSP70 in Murrah and Tarai buffaloes [58] during the summer compared to other seasons (or thermoneutral zone). Moreover, increased expression of *HSP70* in bovine lymphocytes has also been observed after heat stress [59].

Due to subsequent induction of *HSP70* after heat shock and its association with physiological parameters (rectal temperature, pulse, and respiratory rates) of animals [59], serum *HSP70* is considered a sensitive biomarker for heat stress management in livestock [58]. In addition to thermotolerance, studies have also reported other roles of the HSP family in animal physiology. In particular, SNPs identified in the 5′-UTR regions of HSP70 gene have been linked with different performance traits of livestock such as milk production, thermal stress and disease vulnerability [1,43]. Moreover, SNPs have also been identified in the *HSP70* gene of beef cattle breeds (Angus, Brahman, and their crossbreds) with little effect on milk protein and milk fat [60]. Polymorphism (SNP) at the promoter region has been shown to alter the expression level of bovine *HSP70.1* gene, and exhibited its significant association with heat tolerance and higher milk production in Frieswal cattle [43]. These findings indicate the putative effects of selective pressure (natural or artificial selection) on the *HSP70* gene to ultimately favor animals with better thermotolerance, performance, and stress resilience. Our findings are in line with recent whole genome-sequencing studies on buffalo that reported evidence for the selection of genes related to social behavior, digestive physiology, fitness, and milk production [61].

HSPs are highly conserved in nature and play crucial role in stress stimulus and in several normal cellular functions. The genomic characterization of the *HSP* gene family and elucidation of their physiochemical properties performed in the present study would help in better understanding HSPs in buffalo. HSPs are highly conserved proteins and important from evolutionary point of view, so further investigations are warranted to study expression dynamics under various physiological conditions to elucidate their putative roles in thermotolerance and stress physiology in buffalo. In particular, non-synonymous SNPs and indels observed in the present study need to be further explored to detect their effect on protein function and subsequent physiological manifestations in buffalo. These findings might explain the better thermotolerance and fitness of buffalo compared to *Bos taurus*, particularly under tropical climate.

## 5. Conclusions

The present study revealed that the buffalo genome contains 64 genes of the HSP family in buffalo. These genes are widely dispersed across the buffalo genome. Multiple sequence alignment and phylogenetic analysis indicated the highly conserved consensus sequences and evolutionary relatedness of the HSP family. Sequence analysis revealed that the *HSP90* gene family buffalo is more conserved in nature as compared to the *HSP70* family that exhibited a quite higher ratio of non-synonymous substitutions. Structural variations in secondary structures of *HSP40* and *HSP90* were observed in buffalo and cattle. The findings of the present study provide insights into the genomic variations in the HSP gene family that could be used for a better understanding of the function of these genes and their further use for selective breeding of buffalo for better thermotolerance and stress resilience.

## Figures and Tables

**Table 1:** Physiochemical Properties of *HSP70* gene family members.

Sr. No	Gene	Chromosome	Exon Count	MW (kDa)	A.A	pI	AI	II	GRAVY
1	*HSP70.1*	2	2	70.27	641	5.67	85.23	32.84	−0.395
2	*HSPA1L*	2	4	70.36	641	5.89	85.83	32.95	−0.354
3	*HSPA6*	6	1	70.96	643	5.78	83.89	39.18	−0.403
4	*HSPA8*	16	9	71.24	650	5.37	81.02	37.59	−0.454
5	*HSPA2*	11	1	69.81	636	5.5	82.81	35.23	−0.474
6	*HSP70*	1	4	35.79	323	6.11	76.93	35.1	−0.473
7	*HSPA14*	14	16	54.94	509	5.67	95.23	45.33	−0.085
8	*HSPA13*	1	5	51.82	471	5.08	100.13	34.84	−0.111
9	*HSPA4*	9	19	94.46	840	5.13	75.89	45.29	−0.56
10	*HSPA4L*	17	22	94.89	840	5.55	80.44	44.42	−0.539

[MW (Molecular Weight in Kilo Daltons), A.A (number of amino acids), pI (Isoelectric point), AI (Aliphatic Index), II (Instability Index) and GRAVY (Grand Average of hydropathicity Index)].

**Table 2:** Physiochemical Properties of *HSP40* gene family members.

Sr. No.	Gene	Chromosome	Exon Count	MW (kDa)	A.A	pI	AI	II	GRAVY
1	*DNAJA1*	3	9	44.88	397	6.65	71.89	31.57	−0.728
2	*DNAJA4*	20	9	47.97	426	6.63	74.77	39.19	−0.727
3	*DNAJA2*	18	9	45.76	412	6.06	68.08	39.03	−0.699
4	*DNAJB5*	3	4	47.17	420	9.42	69.43	46.44	−0.555
5	*DNAJB4*	6	4	37.82	337	8.66	63.38	43.18	−0.708
6	*DNAJB11*	1	10	40.50	358	5.92	80.25	37.41	−0.568
7	*DNAJB1*	9	6	38.25	340	8.75	63.35	49.59	−0.751
8	*DNAJB13*	16	8	3612	316	7.66	82.03	36.99	−0.509
9	*DNAJA3*	24	12	52.09	480	9.5	66.02	45.15	−0.467
10	*DNAJB8*	21	1	25.23	231	6.46	49	45.52	−0.659
11	*DNAJB6*	8	13	31.13	278	9.31	69.14	47.61	−0.468
12	*DNAJB12*	4	9	41.50	371	8.91	66.31	40.42	−0.722
13	*DNAJB2*	2	10	32.38	294	5.78	55.17	58.66	−0.841
14	*DNAJB3*	6	1	26.83	244	6.07	53.16	50.15	−0.616
15	*DNAJB9*	8	3	25.83	223	8.68	41.61	39.56	−0.861
16	*DNAJB14*	7	11	36.85	329	8.76	59.27	35.91	−0.874
17	*DNAJC5*	14	8	22.13	198	4.93	65.15	34.45	−0.476
18	*DNAJB7*	4	2	25.21	217	7.03	44.01	39.69	−1.102
19	*DNAJC5G*	12	10	20.87	186	7.92	63.55	40.85	−0.534
20	*DNAJC5B*	15	8	22.69	199	5.62	67.19	50.49	−0.474
21	*DNAJC18*	9	8	41.68	358	8.28	65.42	42.81	−0.892
22	*DNAJC21*	19	12	61.67	533	5.45	53.15	50.3	−1.251
23	*DNAJC16*	5	16	91.94	794	6.82	86.18	40.41	−0.346
24	*DNAJC10*	2	24	91.30	793	6.57	77.36	36.89	−0.419
25	*DNAJC17*	11	11	34.60	304	6.96	78.39	46.35	−0.906
26	*DNAJC3*	13	13	57.70	504	5.64	78.83	44.97	−0.677
27	*DNAJC30*	24	2	25.87	226	10.64	68.76	60.79	−0.76
28	*DNAJC11*	5	16	63.22	559	8.5	87.75	47.33	−0.405
29	*DNAJC1*	14	12	62.91	543	8.98	81.05	63.77	−0.833
30	*DNAJC7*	3	15	56.45	494	6.56	64.76	41.63	−0.722
31	*DNAJC4*	5	8	26.93	236	10.6	65.42	69.46	−0.856
32	*DNAJC14*	4	8	78.06	699	8.34	67.73	51.48	−0.614
33	*DNAJC24*	16	5	17.20	149	4.69	78.59	59.41	−0.587
34	*DNAJC9*	4	5	34.30	300	6.11	70.27	49.68	−0.785
35	*DNAJC27*	12	7	30.73	273	8.55	75.71	33.54	−0.421
36	*DNAJC25*	3	4	42.55	359	9.21	84.54	62.04	−0.627
37	*DNAJC13*	1	59	254.36	2243	6.35	92.76	42.37	−0.234
38	*DNAJC12*	4	6	23.23	198	5.11	57.17	73.75	−1.024
39	*DNAJC15*	13	6	15.89	149	10.1	88.59	48.02	−0.182

[MW (Molecular Weight in Kilo Daltons), A.A (number of amino acids), pI (Isoelectric point), AI (Aliphatic Index), II (Instability Index) and GRAVY (Grand Average of hydropathicity Index)].

**Table 3:** Physiochemical Properties of HSP family B, *HSP10*, *HSPD*, and *HSPH1*.

Sr. No	Gene	Chromosome	Exon Count	MW (kDa)	A.A	pI	AI	II	GRAVY
1	*HSPB1*	24	3	22.39	201	5.98	70.4	61.45	−0.591
2	*HSPB2*	16	2	20.20	182	5.07	78.24	46.26	−0.505
3	*HSPB3*	19	1	16.73	149	5.33	96.24	47.66	−0.217
4	*HSPB6*	18	3	17.49	164	5.95	89.94	63.95	−0.121
5	*HSPB7*	2	3	18.23	167	5.6	60.84	59.89	−0.501
6	*HSPB8*	17	3	21.67	196	5.13	59.13	72.61	−0.549
7	*HSPB9*	3	1	16.87	157	8.56	68.28	46.32	−0.38
8	*HSPB11*	6	11	20.14	176	6.35	87.44	46.75	−0.16
9	*HSPD1*	2	13	60.98	573	5.71	100.91	30.59	−0.087
10	*HSP10*	2	12	10.93	102	8.89	95.39	23.75	−0.041
11	*HSPH1*	13	18	91.69	817	5.32	78.4	40.77	−0.525

[MW (Molecular Weight in Kilo Daltons), A.A (number of amino acids), pI (Isoelectric point), AI (Aliphatic Index), II (Instability Index) and GRAVY (Grand Average of hydropathicity Index)].

**Table 4:** Physiochemical Properties of *HSP90* gene family members.

Sr. No	Gene	Chromosome	Exon Count	MW (kDa)	A.A	pI	AI	II	GRAVY
1	*HSP90AA1*	20	11	84.74	733	4.93	79.4	43.03	−0.751
2	*HSP90AB1*	2	12	83.25	724	4.96	81.05	42.3	−0.679
3	*HSP90B1*	4	18	92.43	804	4.76	76.63	40.41	−0.722
4	*TRAP1*	24	18	79.53	703	7.3	90.48	44.82	−0.336

[MW (Molecular Weight in Kilo Daltons), A.A (number of amino acids), pI (Isoelectric point), AI (Aliphatic Index), II (Instability Index) and GRAVY (Grand Average of hydropathicity Index)].

**Table 5:** Ten differentially conserved motifs of heat-shock protein gene family observed in buffalo.

Motif	Protein Sequence	Length	Pfam Domain
MEME-1	DYYEILGVPRGASDEEIKKAYRKLALKYHPDKNPDPGAEAE	41	DnaJ
MEME-2	FKZIAEAYEVLSDPEKRKIYDKYGEEGL	28	DnaJ
MEME-3	IDLGTTYSCVGVFQHGKVEIIANDQGBRTTPSYVAFTDTERLIGDAAKNQ	50	Hsp70
MEME-4	PVTBAVITVPAYFNDSQRQATKDAGQIAGLNVLRJINEPTAAAJAYGJDK	50	Hsp70
MEME-5	TAGDTHLGGEDFDNRLVNHFCEEFKRKHKKDISENKRALRRLRTACERAK	50	Hsp70
MEME-6	TAGGVMTVLIKRNSTIPTKQTQTFTTYSDNQPGVLIQVYEGERAMTKDNN	50	Hsp70
MEME-7	GIPPAPRGVPQIEVTFDIDANGILNVTAVDKSTGKENKITITNDKGRLSK	50	Hsp70
MEME-8	DFFNGKELNKSINPDEAVAYGAAVQAAIL	29	Hsp70
MEME-9	TLSSSTQAPLEIDSLYEGIDFYTSITRARFEELCADLFRGT	41	Hsp70
MEME-10	LDKCQEVINWLDKNQLAEKEEFEHKQKELEQVCNPIISKLY	41	-

**Table 6:** SNPs in coding region of *HSP 70* and *HSP90* gene family in buffalo.

Gene	Synonymous (ks)	Non-synonymous (ka)	Total	Ka/Ks Ratio
***HSP70* gene family**				
*HSPA4L*	**18**	**6**	24	0.33
*HSPA4*	13	3	16	0.23
*HSPA13*	24	9	33	0.38
*HSPA14*	8	2	10	0.25
*HSP70*	39	23	62	0.59
*HSPA8*	72	8	80	0.11
*HSP70.1*	17	1	18	0.06
*HSPA1L*	22	3	25	0.14
*HSPA6*	19	4	23	0.21
**Total**	**232**	**59**	**291**	**0.25**
**HSP90 gene family**				
*TRP1*	111	17	128	0.15
*HSP90AA1*	23	1	24	0.04
*HSP90B1*	19	0	19	0.00
*HSP90AB1*	22	1	23	0.05
**Total**	**175**	**19**	**194**	**0.11**

## Supplementary Materials

The following are available online at https://www.mdpi.com/2073-4425/11/11/1388/s1, Figure S1: Tree with alignment view of *HSP40*. Percentage of sequence is represented with different colors in Identity bar at top of the sequences green color indicates 100% identity, green-brown shows at least 30 and under 100% while red representing below 30 identity. Figure S2: Tree with alignment view of *HSP70*. Percentage of sequence is represented with different colors in Identity bar at top of the sequences green color indicates 100% identity, green-brown shows at least 30 and under 100% while red representing below 30 identity. Figure S3: Tree with alignment view of *HSP90*. Percentage of sequence is represented with different colors in Identity bar at top of the sequences green color indicates 100% identity, green-brown shows at least 30 and under 100% while red representing below 30 identity. Figure S4: Tree with alignment view of *HSPD*. Percentage of sequence is represented with different colors in Identity bar at top of the sequences green color indicates 100% identity, green-brown shows at least 30 and under 100% while red representing below 30 identity. Figure S5: Tree with alignment view of *HSP10*. Percentage of sequence is represented with different colors in Identity bar at top of the sequences green color indicates 100% identity, green-brown shows at least 30 and under 100% while red representing below 30 identity. Figure S6: Tree with alignment view of *HSPH1*. Percentage of sequence is represented with different colors in Identity bar at top of the sequences green color indicates 100% identity, green-brown shows at least 30 and under 100% while red representing below 30 identity. Figure S7: Tree with alignment view of *HSPB*. Percentage of sequence is represented with different colors in Identity bar at top of the sequences green color indicates 100% identity, green-brown shows at least 30 and under 100% while red representing below 30 identity. Table S1: Accession no. of different heat-shock protein in different organisms used in phylogenetic analysis, Table S2: Accession no. of buffalo *HSP90*, Table S3: Accession no. of buffalo *HSP70*, Table S4: Accession no. of buffalo HSP40, Table S5: Accession no. of buffalo *HSP family B*, *HSP10*, *HSPD*, and *HSPH1*, Table S6: Mutations in coding region of HSP gene family in buffalo.
