# Supplementary material for: Genomic Identification, Evolution and Sequence Analysis of the Heat-Shock Protein Gene Family in Buffalo
Source: Genes (Basel). 2020 Nov 23;11(11):1388. doi: 10.3390/genes11111388 (PMC7700627; doi:10.3390/genes11111388)
Supplement: Supplementary file 1 [file genes-11-01388-s001.pdf]

genes

MDPI

Article

Genomic Identification, Evolution and Sequence Analysis of the Heat-Shock Protein Gene Family in Buffalo

Saif ur Rehman [1,†], Asif Nadeem [2,†], Maryam Javed [3], Faiz-ul Hassan [4,*], Xier Luo [1], Ruqayya Bint Khalid [3] and Qingyou Liu [1,*]

1 State Key Laboratory for Conservation and Utilization of Subtropical Agro-Bioresources, Guangxi University, Nanning, Guangxi, 530005, China; saif_ali28@yahoo.com (S.u.R.); luoxier92@163.com (X.L.)
2 Department of Biotechnology, Virtual University of Pakistan, Lahore-54000, Pakistan; asif.nadeem@vu.edu.pk (A.N.)
3 Institute of Biochemistry and Biotechnology, University of Veterinary and Animal Sciences, Lahore, Pakistan; Maryam.javed@uvas.edu.pk (M.J.); ruqiakhalid0@gmail.com (R.B.K.)
4 Institute of Animal and Dairy Sciences, Faculty of Animal Husbandry, University of Agriculture, Faisalabad-38040, Pakistan
* Correspondence: f.hassan@uaf.edu.pk (F.-u.H.); qyliu-gene@gxu.edu.cn (Q.L.); Tel.: +86-138-7880-5296 (Q.L.)
† These authors contributed equally to this manuscript.

Received: 26 October 2020; Accepted: 18 November 2020; Published: 23 November 2020

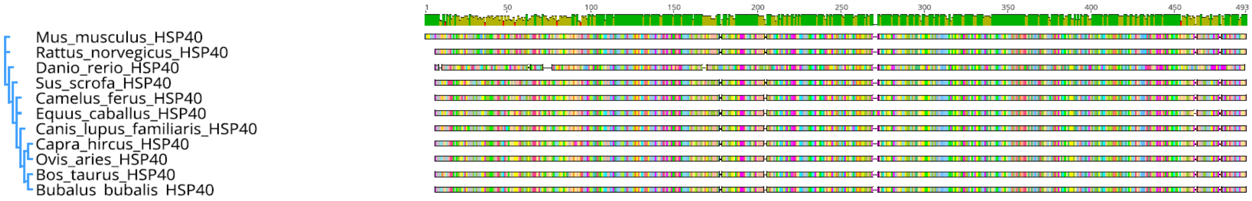

Figure S1. Tree with alignment view of HSP40. Percentage of sequence is represented with different colors in Identity bar at top of the sequences green color indicates 100% identity, green-brown shows at least 30 and under 100% while red representing below 30 identity.

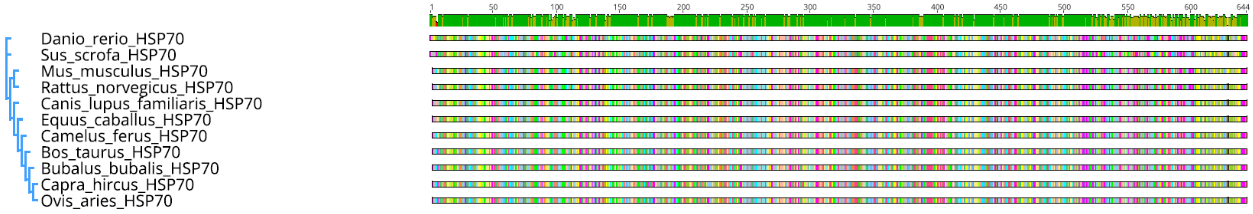

Figure S2. Tree with alignment view of HSP70. Percentage of sequence is represented with different colors in Identity bar at top of the sequences green color indicates 100% identity, green-brown shows at least 30 and under 100% while red representing below 30 identity.

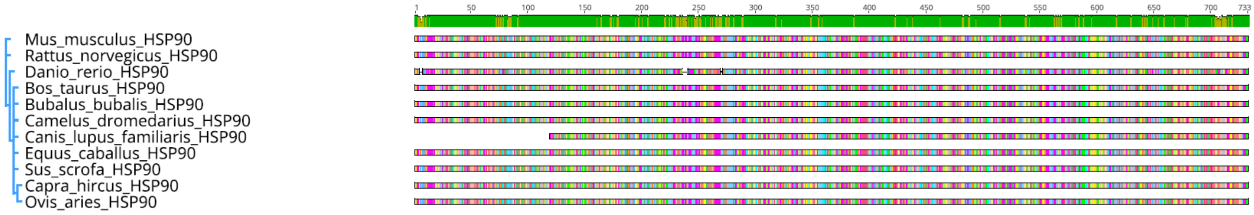

Figure S3. Tree with alignment view of HSP90. Percentage of sequence is represented with different colors in Identity bar at top of the sequences green color indicates 100% identity, green-brown shows at least 30 and under 100% while red representing below 30 identity.

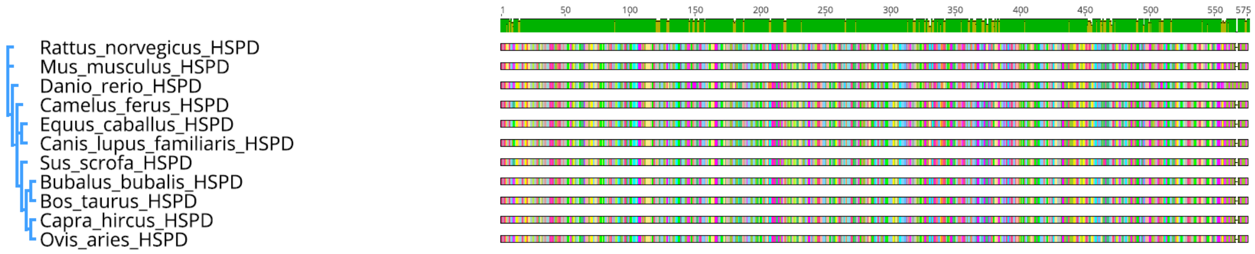

Figure S4. Tree with alignment view of HSPD. Percentage of sequence is represented with different colors in Identity bar at top of the sequences green color indicates 100% identity, green-brown shows at least 30 and under 100% while red representing below 30 identity.

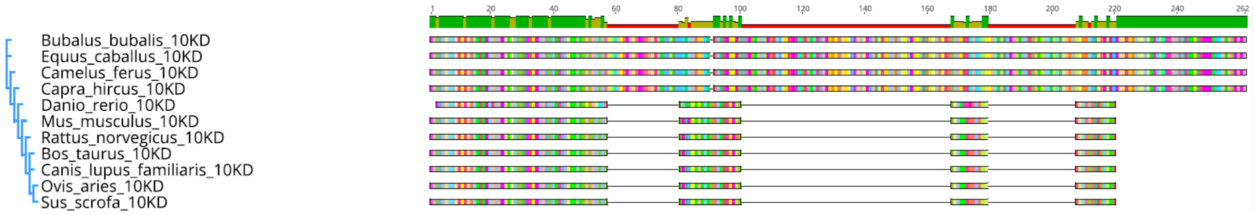

Figure S5. Tree with alignment view of HSP10. Percentage of sequence is represented with different colors in Identity bar at top of the sequences green color indicates 100% identity, green-brown shows at least 30 and under 100% while red representing below 30 identity.

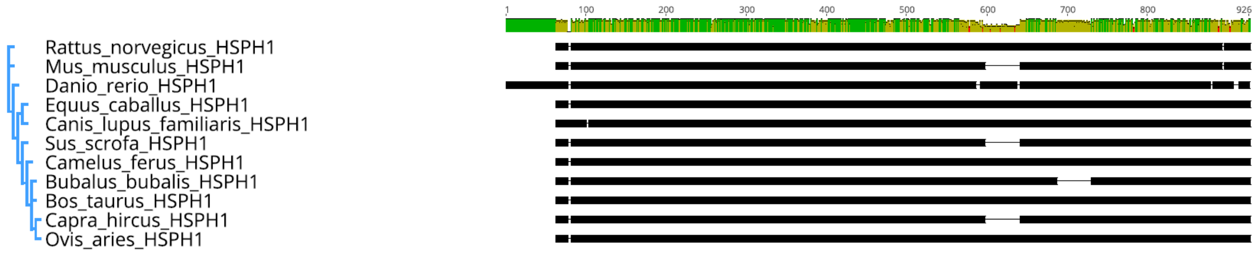

Figure S6. Tree with alignment view of HSPH1. Percentage of sequence is represented with different colors in Identity bar at top of the sequences green color indicates 100% identity, green-brown shows at least 30 and under 100% while red representing below 30 identity.

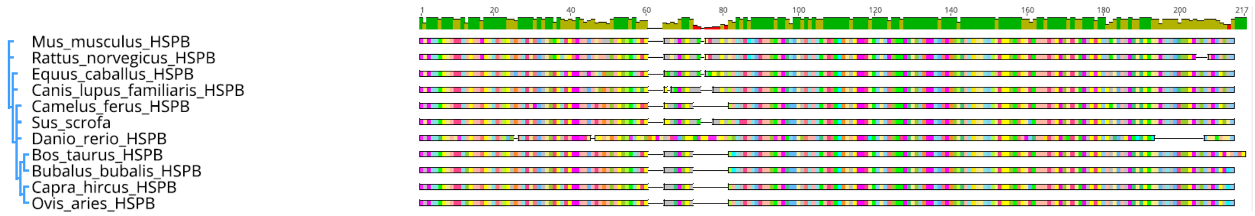

Figure S7. Tree with alignment view of HSPB. Percentage of sequence is represented with different colors in Identity bar at top of the sequences green color indicates 100% identity, green-brown shows at least 30 and under 100% while red representing below 30 identity.

Table S1. Accession no. of different heat shock protein in different organisms used in phylogenetic analysis

Sr. No.	HSP Gene	Accession No.
1	Capra hircus HSP10	XP_017916787.1
2	Ovis aries HSP10	XP_027820863.1 10
3	Equus caballus HSP10	XP_023478058.1
4	Bos taurus HSP10	NP_776771.1 10
5	Bubalus bubalis HSP10	XP_025133715.1
6	Camelus ferus HSP10	XP_006175275.2
7	Sus scrofa HSP10	NP_999472.1
8	Canis lupus familiaris HSP10	XP_852207.1 10
9	Mus musculus HSP10	NP_032329.1
10	Rattus norvegicus HSP10	NP_037098.1 10
11	Danio rerio HSP10	NP_001315319.1 10
12	Capra hircus HSP40	XP_005697986.3
13	Ovis aries HSP40	XP_014959545.1
14	Equus caballus HSP40	XP_014585675.1
15	Bos Taurus HSP40	XP_005224490.1
16	Bubalus bubalis HSP40	XP_006054938.1
17	Camelus ferus HSP40	XP_032317011.1
18	Sus scrofa HSP40	XP_003354675.1
19	Canis lupus familiaris HSP40	XP_851751.1
20	Mus musculus HSP40	EDK97241.1
21	Rattus norvegicus HSP40	NP_001033684.1
22	Danio rerio HSP40	AAH55555.1
23	Capra hircus HSP70	NP_001272632.1
24	Ovis aries HSP70	AEX55801.1
25	Equus caballus HSP70	NP_001243852.1
26	Bos taurus HSP70	NP_976067.3
27	Bubalus bubalis HSP70	XP_006041955.2
28	Camelus ferus HSP70	XP_032318576.1
29	Sus scrofa HSP70	NP_001116599.1
30	Canis lupus familiaris HSP70	NP_001003067.2
31	Mus musculus HSP70	NP_034608.2
32	Rattus norvegicus HSP70	NP_001316825.1
33	Danio rerio HSP70	AAH56709.1
34	Capra hircus HSP90	XP_017921728.1

35	Ovis aries HSP90	ABI99473.1
36	Equus caballus HSP90	NP_001157427.1
37	Bos taurus HSP90	NP_001012688.1
38	Bubalus bubalis HSP90	XP_025127285.1
39	Camelus ferus HSP90	XP_031310187.1
40	Sus scrofa HSP90	NP_999138.1
41	Canis lupus familiaris HSP90	XP_005623992.2
42	Mus musculus HSP90	NP_034610.1
43	Rattus norvegicus HSP90	NP_786937.1
44	Danio rerio HSP90	NP_571403.1
45	Capra hircus HSPB	XP_017896392.1
46	Ovis aries HSPB	XP_027817273.1
47	Equus caballus HSPB	XP_001504528.1
48	Bos taurus HSPB	NP_001020740.1
49	Bubalus bubalis HSPB	XP_006057191.1
50	Camelus ferus HSPB	XP_032315487.1
51	Sus scrofa HSPB	NP_001007519.1
52	Canis lupus familiaris HSPB	NP_001003295.2
53	Mus musculus HSPB	NP_038588.2
54	Rattus norvegicus HSPB	NP_114176.4
55	Danio rerio HSPB	NP_001008615.2
56	Capra hircus HSPD	XP_017916768.1
57	Ovis aries HSPD	XP_027820862.1
58	Equus caballus HSPD	XP_023478054.1
59	Bos taurus HSPD	NP_001160081.1
60	Bubalus bubalis HSPD	XP_006078223.1
61	Camelus ferus HSPD	XP_032336014.1
62	Sus scrofa HSPD	NP_001241645.1
63	Canis lupus familiaris HSPD	XP_022270952.1
64	Mus musculus HSPD	NP_034607.3
65	Rattus norvegicus HSPD	XP_006244994.1
66	Danio rerio HSPD	NP_851847.1
67	Capra hircus HSPH1	XP_017912110.1
68	Ovis aries HSPH1	XP_014953627.2
69	Equus caballus HSPH1	XP_001493567.1
70	Bos taurus HSPH1	NP_001068770.1
71	Bubalus bubalis HSPH1	XP_025118688.1
72	Camelus ferus HSPH1	XP_032352233.1
73	Sus scrofa HSPH1	XP_020920751.1

74	Canis lupus familiaris HSPH1	XP_013962905.1
75	Mus musculus HSPH1	NP_001334463.1
76	Rattus norvegicus HSPH1	NP_001011901.1
77	Danio rerio HSPH1	XP_001919957.2

Table S2. Accession no. of buffalo HSP90

**Sr. No**	**Gene**	**Accession Number**
1	HSP90AA1	XP_025127285.1
2	HSP90AB1	XP_006069362.2
3	HSP90B1	XP_006077264.1
4	TRAP1	XP_006054992.2

Table S3. Accession no. of buffalo HSP70

**Sr. No**	**Gene**	**Accession Number**
1	HSP70.1	XP_006041955.2
2	HSPA1L	XP_006041950.1
3	HSPA6	XP_006041196.2
4	HSPA8	XP_006072590.1
5	HSPA2	XP_006072201.1
6	HSP70	XP_025120572.1
7	HSPA14	XP_006066069.1
8	HSPA13	XP_006078343.1
9	HSPA4	XP_006065134.1
10	HSPA4L	XP_025123823.1

Table S4. Accession no. of buffalo HSP40

**Sr. No**	**Gene**	**Accession Number**
1	DNAJA1	XP_006066768.1
2	DNAJA4	XP_025127429.1
3	DNAJA2	XP_006062820.1
4	DNAJB5	XP_025137445.1
5	DNAJB4	XP_006068502.1
6	DNAJB11	XP_006058686.1
7	DNAJB1	XP_006066611.1
8	DNAJB13	XP_025122222.1
9	DNAJA3	XP_006054938.1
10	DNAJB8	XP_006078907.1
11	DNAJB6	XP_025147736.1

12	DNAJB12	XP_006077137.2
13	DNAJB2	XP_006059040.1
14	DNAJB3	XP_006053793.1
15	DNAJB9	XP_006050765.1
16	DNAJB14	XP_006052550.1
17	DNAJC5	XP_025119663.1
18	DNAJB7	XP_025138593.1
19	DNAJC5G	XP_006046244.2
20	DNAJC5B	XP_025121114.1
21	DNAJC18	XP_006070872.1
22	DNAJC21	XP_006071958.1
23	DNAJC16	XP_006072848.2
24	DNAJC10	XP_006071841.1
25	DNAJC17	XP_006073122.1
26	DNAJC3	XP_006074344.1
27	DNAJC30	XP_006057157.2
28	DNAJC11	XP_006044303.1
29	DNAJC1	XP_025120211.1
30	DNAJC7	XP_006055827.1
31	DNAJC4	XP_025142383.1
32	DNAJC14	XP_006075140.1
33	DNAJC24	XP_006060883.1
34	DNAJC9	XP_006060667.1
35	DNAJC27	XP_006055128.1
36	DNAJC25	XP_006043444.2
37	DNAJC13	XP_025147498.1
38	DNAJC12	XP_006071587.1
39	DNAJC15	XP_025118814.1

Table S5. Accession no. of buffalo HSP family B, HSP10, HSPD and HSPH1

Sr. No	Gene	Accession Number
1	HSPB1	XP_006057191.1
2	HSPB2	XP_025122686.1
3	HSPB3	XP_006074232.1
4	HSPB6	XP_006068849.1
5	HSPB7	XP_006066657.1
6	HSPB8	XP_006061021.1
7	HSPB9	XP_025135916.1
8	HSPB11	XP_006067277.1
9	HSPD1	XP_006078223.1

10	HSP10	XP_006078224.1
11	HSPH1	XP_025118688.1

Table S6. Mutations in coding region of HSP gene family in buffalo

Gene	Nucleotide variation	Amino acid change	Mutation type
**HSP90AA1**	T108>G	T36	Synonymous
	C166>T	L56	Synonymous
	C201>A	P67	Synonymous
	T213>C	D71	Synonymous
	C240>A	L80	Synonymous
	A516>G	V172	Synonymous
	C768>T	P256	Synonymous
	T930>C	Y310	Synonymous
	T978>G	A326	Synonymous
	T1059>C	F353	Synonymous
	C1125>T	C375	Synonymous
	T1185>C	L395	Synonymous
	G1518>A	S506	Synonymous
	C1575>A	P525	Synonymous
	T1656G	L552	Synonymous
	G1746>A	K582	Synonymous
	T1800>C	I600	Synonymous
	G1851>T	A617	Synonymous
	T2121>G	D707>E	Non-Synonymous
	A2154>G	P718	Synonymous
	A2163>G	E721	Synonymous
	A2166>G	G722	Synonymous
	A2193>G	E731	Synonymous
**HSP90AB1**	A153>G	L51	Synonymous
	G336>A	A112	Synonymous
	G567>A	Q189	Synonymous
	C1158>T	L386	Synonymous
	A1260>G	A420	Synonymous
	A1284>G	K428	Synonymous
	T1290>C	A430	Synonymous
	G1296>A	A432	Synonymous
	C1308>T	N436	Synonymous
	G1314>A	K438	Synonymous
	G1437>C	T479	Synonymous

	T1491>C	S497	Synonymous
	A1542>G	T514	Synonymous
	C1575>G	L525	Synonymous
	A1599>G	L533	Synonymous
	T1638>C	D546	Synonymous
	T1752>C	L584	Synonymous
	C1788>T	Y596	Synonymous
	T1875>C	H625	Synonymous
	T2070>C	I690	Synonymous
	G2082>A	E694	Synonymous
	T2103>C	S701	Synonymous
**HSP90B1**	C69>T	D23	Synonymous
	T438>C	H146	Synonymous
	A510>G	G170	Synonymous
	A564>G	T188	Synonymous
	C576>T	I192	Synonymous
	T600>C	Y200	Synonymous
	A660>G	Q220	Synonymous
	T699>C	I233	Synonymous
	C858>T	T286	Synonymous
	T879>C	A293	Synonymous
	T1218>C	S406	Synonymous
	C1260>T	D420	Synonymous
	C1548>T	H516	Synonymous
	C1647>T	A549	Synonymous
	C1752>T	D584	Synonymous
	G2187>A	L729	Synonymous
	T2289>C	P763	Synonymous
	T2310>C	T770	Synonymous
	C2361>T	D787	Synonymous
**TRP1**	G9>C	R3	Synonymous
	T20>C	M7>T	Non-Synonymous
	G30>T	L10	Synonymous
	G32>C	W11>S	Non-Synonymous
	G33>T	W11>S	Non-Synonymous
	G73>A	G25>R	Non-Synonymous
	C117>T	S39	Synonymous
	C128>G	P43>R	Non-Synonymous
	T230>C	L77>S	Non-Synonymous
	C236>T	A79>V	Non-Synonymous

	T237>C	A79>V	Non-Synonymous
	C288>G	L96	Synonymous
	G355>T	A119>S	Non-Synonymous
	C429>T	H143	Synonymous
	G555>A	A185	Synonymous
	G570>A	A190	Synonymous
	G660>C	S220	Synonymous
	G732>A	S244	Synonymous
	T837>C	F279	Synonymous
	T879>C	T293	Synonymous
	G916>A	G306>S	Non-Synonymous
	C918>T	G306>S	Non-Synonymous
	T951>C	V317	Synonymous
	G996>A	T332	Synonymous
	C1142>T	T381>M	Non-Synonymous
	C1277>T	T426>I	Non-Synonymous
	G1313>A	R438>K	Non-Synonymous
	C1389>T	I463	Synonymous
	G1392>A	A464	Synonymous
	T1428>C	A476	Synonymous
	T1447>C	S483>P	Non-Synonymous
	G1449>A	S483>P	Non-Synonymous
	C1455>T	Y485	Synonymous
	C1473>T	A491	Synonymous
	T1500>C	C500	Synonymous
	G1521>A	A507	Synonymous
	A1653>G	T551	Synonymous
	C1703	P568	Indel
	A1704	P568	Indel
	G1705	A569	Indel
	T1714>C	C572>R	Non-Synonymous
	T1761>C	N587	Synonymous
	G1764>A	A588	Synonymous
	G1830>A	T610	Synonymous
	T1848>C	A616	Synonymous
	A1938>G	R646	Synonymous
	G2085>T	L695	Synonymous
**HSP70.1**	G156>C	G52	Synonymous
	A237>C	G79	Synonymous
	C282>G	V94	Synonymous

	A324>G	K108	Synonymous
	C393>G	A131	Synonymous
	A603>C	G201	Synonymous
	A798>G	A266	Synonymous
	A816>G	R272	Synonymous
	C930>T	F310	Synonymous
	G951>A	V317	Synonymous
	G960>T	A320	Synonymous
	T1177>C	L393	Synonymous
	G1581>A	A527	Synonymous
	G1632>T	S544	Synonymous
	C1746>T	D582	Synonymous
	T1866>C	F622	Synonymous
	G1877>C	G626>A	Non- Synonymous
**HSPA1L**	A9>G	A3	Synonymous
	T24>C	A8	Synonymous
	T57>C	C19	Synonymous
	G60>A	V20	Synonymous
	A72>G	Q24	Synonymous
	C129>T	Y43	Synonymous
	T141>C	T47	Synonymous
	A288>G	V96	Synonymous
	A357>G	E119	Synonymous
	G489>A	A163	Synonymous
	C660>T	E220	Synonymous
	A738>G	E246	Synonymous
	G1029>T	T343	Synonymous
	G1149>A	M383>A	Non- Synonymous
	G1195>T	A399>S	Non- Synonymous
	T1243>C	L415	Synonymous
	T1299>C	Y433	Synonymous
	T1356>G	T452	Synonymous
	T1422>C	G474	Synonymous
	C1470>A	V490	Synonymous
	C1650>T	N550	Synonymous
	T1742>C	L581>S	Non- Synonymous
**HSPA6**	T69>C	F23	Synonymous
	A237>G	R79	Synonymous
	T250>A	S84>T	Non- Synonymous
	T348>C	F116	Synonymous

	A519>G	R173	Synonymous
	C756>G	G252	Synonymous
	C894>T	S298	Synonymous
	T936>C	F312	Synonymous
	A957>G	V319	Synonymous
	C972>G	R324	Synonymous
	G1069>A	D357>N	Non- Synonymous
	A1143>G	V381	Synonymous
	T1179>G	L393	Synonymous
	G1209>A	L403	Synonymous
	T1335>C	Y445	Synonymous
	G1709>A	R570>H	Non- Synonymous
	T1855>C	S619>P	Non- Synonymous
	C1878>T	A626	Synonymous
	C1917>T	I639	Synonymous
**HSPA8**	C63>T	F21	Synonymous
	G147>A	R49	Synonymous
	A150>G	L50	Synonymous
	A162>T	A54	Synonymous
	A165>C	A55	Synonymous
	A360>T	S120	Synonymous
	C429>T	V143	Synonymous
	C504>T	N168	Synonymous
	A538>G	T180>A	Non- Synonymous
	C582>T	N194	Synonymous
	G603>T	G201	Synonymous
	A660>G	K220	Synonymous
	T714>C	V238	Synonymous
	G729>A	A243	Synonymous
	G804>A	E268	Synonymous
	A975>G	K325	Synonymous
	T1026>C	R342	Synonymous
	T1254>C	T418	Synonymous
	C1317>T	L439	Synonymous
	G1386>A	T462	Synonymous
	C1575>T	Y525	Synonymous
	C1602>T	D534	Synonymous
	T1611>C	S537	Synonymous
	G1623>A	S541	Synonymous
	C1635>T	Y545	Synonymous

	G1695>A	E565	Synonymous
	T1893>A	G631	Synonymous
**HSPA2**	C33>T	D11	Synonymous
	C48>T	Y16	Synonymous
	G159>C	G53	Synonymous
	A160>G	T54>D	Non- Synonymous
	C161>A	T54>D	Non- Synonymous
	G312>A	V104	Synonymous
	C537>G	A179	Synonymous
	C540>G	A180	Synonymous
	T555>C	G185	Synonymous
	T681>C	G227	Synonymous
	T730>C	W244>L	Non- Synonymous
	G731>T	W244>L	Non- Synonymous
	G775>A	N259	Synonymous
	C776>A	N259	Synonymous
	C777	N259	Indel
	A778	K260	Indel
	A779	K260	Indel
	G780	K260	Indel
	C781	R261	Indel
	G782	R261	Indel
	C783	R261	Indel
	G784	A262	Indel
	C785	A262	Indel
	G786	A262	Indel
	G787	V263	Indel
	T788	V263	Indel
	G789	V263	Indel
	C790	R264	Indel
	G791	R264	Indel
	A814>C	R272	Synonymous
	G816>T	R272	Synonymous
	T894>G	T298	Synonymous
	T944>G	V315>G	Non- Synonymous
	A945>G	V315>G	Non- Synonymous
	C946>A	P316>T	Non- Synonymous
	T1107>C	D369	Synonymous
	A1113>G	A371	Synonymous
	T1152>C	I384	Synonymous

	T1170>C	N390	Synonymous
	G1533>T	G511	Synonymous
	T1534>C	C512>R	Non- Synonymous
	A1575>G	A525	Synonymous
	T1650>C	Y550	Synonymous
	C1848>T	G616	Synonymous
	C1881>T	S627	Synonymous
**HSP70**	G25>A	V9>I	Non- Synonymous
	A156>C	G52	Synonymous
	G183>C	L61	Synonymous
	A220>C	I74>L	Non- Synonymous
	C265>G	H89>E	Non- Synonymous
	C267>G	H89>E	Non- Synonymous
	A316>C	S106>R	Non- Synonymous
	G324>A	K108	Synonymous
	A373>C	T125>P	Non- Synonymous
	A382>C	K128>H	Non- Synonymous
	G384>C	K128>H	Non- Synonymous
	G489>C	V163	Synonymous
	A502>T	N168>Y	Non- Synonymous
	A511>G	R171>G	Non- Synonymous
	A658>C	K220>Q	Non- Synonymous
	G689>A	G230>E	Non- Synonymous
	A704>G	N235>S	Non- Synonymous
	A715>G	N239>D	Non- Synonymous
	A781>C	R261	Synonymous
	G865>C	E289>Q	Non- Synonymous
	G898>T	A300>S	Non- Synonymous
	G942>A	L314	Synonymous
	A995>G	H332>R	Non- Synonymous
	C1062>A	F354>L	Non- Synonymous
	C1086>A	S362>R	Non- Synonymous
	G1140>A	L380	Synonymous
	G1146>C	G382	Synonymous
	A1240>G	I414>V	Non- Synonymous
	A1318>G	V438	Synonymous
	A1383>G	L461	Synonymous
	G1428>C	V476	Synonymous
	A1459>T	N487>Y	Non- Synonymous
	A1531>G	S511>G	Non- Synonymous

	C1593>G	V531	Synonymous
	T1737>C	S579	Synonymous
	C1746>T	D582	Synonymous
	T1761>G	A587	Synonymous
	T1866>C	F622	Synonymous
	G1877>C	G626>A	Non- Synonymous
**HSPA14**	G255>A	T85	Synonymous
	T295>C	L99	Synonymous
	G307>A	D103>N	Non- Synonymous
	G519>A	P173	Synonymous
	G552>A	Q184	Synonymous
	C686>T	T229>I	Non- Synonymous
	C715>T	L239	Synonymous
	T723>A	L241	Synonymous
**HSPA13**	G123>C	T41	Synonymous
	C228>T	D76	Synonymous
	C519>T	S173	Synonymous
	A582>C	G194	Synonymous
	C615>A	T205	Synonymous
	T681>C	G227	Synonymous
	A708>G	L236	Synonymous
	G783>A	Q261	Synonymous
	A861>C	R287	Synonymous
	A919>T	M307>L	Non- Synonymous
	C928>T	M310	Synonymous
	A988>G	K330>E	Non- Synonymous
	T997>C	F333>L	Non- Synonymous
	A1004>C	Q335>P	Non- Synonymous
	A1009>G	N337>D	Non- Synonymous
	C1029>T	S343	Synonymous
	A1030>G	M344>V	Non- Synonymous
	A1047>T	L349	Synonymous
	A1060>C	N354>R	Non- Synonymous
	A1061>G	N354>R	Non- Synonymous
	C1113>T	D371	Synonymous
	A1114>G	T372>A	Non- Synonymous
	T1146>C	L382	Synonymous
	C1152>T	P384	Synonymous
**HSPA4**	G429>A	S143	Synonymous
	A469>G	M157>V	Non- Synonymous

	C684>T	D228	Synonymous
	A951>G	V317	Synonymous
	A960>G	P320	Synonymous
	C1104>T	A368	Synonymous
	C1116>T	V372	Synonymous
	A1194>G	V398	Synonymous
	A1452>G	V484	Synonymous
	G2123>A	Q708	Synonymous
	C2269>T	L757	Synonymous
	T2375>C	L792>P	Non- Synonymous
	A2475>G	A825	Synonymous
**HSPA4L**	T45>C	I15	Synonymous
	A633>G	S211	Synonymous
	G678>C	T226	Synonymous
	A984>T	A328	Synonymous
	T1197>C	P399	Synonymous
	G1349>A	R450>H	Non- Synonymous
	G1521>C	Q507>H	Non- Synonymous
	T1530>C	V510	Synonymous
	C1531>G	P511>A	Non- Synonymous
	C1667>A	T556>N	Non- Synonymous
	C1791>T	L597	Synonymous
	C1944>T	D648	Synonymous
	C2221>T	H741>Y	Non- Synonymous
	A2316>C	I772	Synonymous
	C2337>G	L779	Synonymous
	C2355>T	P785	Synonymous
	G2414>A	S805>N	Non- Synonymous
	C2424>T	N808	Synonymous

Publisher's Note: MDPI stays neutral with regard to jurisdictional claims in published maps and institutional affiliations.

© 2020 by the authors. Licensee MDPI, Basel, Switzerland. This article is an open access article distributed under the terms and conditions of the Creative Commons Attribution (CC BY) license (http://creativecommons.org/licenses/by/4.0/).
